# Development and Characterization of 1,827 Expressed Sequence Tag-Derived Simple Sequence Repeat Markers for Ramie (*Boehmeria nivea* L. Gaud)

**DOI:** 10.1371/journal.pone.0060346

**Published:** 2013-04-02

**Authors:** Touming Liu, Siyuan Zhu, Lili Fu, Qingming Tang, Yongting Yu, Ping Chen, Mingbao Luan, Changbiao Wang, Shouwei Tang

**Affiliations:** 1 Institute of Bast Fiber Crops and Center of Southern Economic Crops, Chinese Academy of Agricultural Sciences, Changsha, China; 2 Biotechnology Research Center, Shanxi Academy of Agricultural Sciences, Taiyuan, China; University of Delhi South Campus, India

## Abstract

Ramie (*Boehmeria nivea* L. Gaud) is one of the most important natural fiber crops, and improvement of fiber yield and quality is the main goal in efforts to breed superior cultivars. However, efforts aimed at enhancing the understanding of ramie genetics and developing more effective breeding strategies have been hampered by the shortage of simple sequence repeat (SSR) markers. In our previous study, we had assembled *de novo* 43,990 expressed sequence tags (ESTs). In the present study, we searched these previously assembled ESTs for SSRs and identified 1,685 ESTs (3.83%) containing 1,878 SSRs. Next, we designed 1,827 primer pairs complementary to regions flanking these SSRs, and these regions were designated as SSR markers. Among these markers, dinucleotide and trinucleotide repeat motifs were the most abundant types (36.4% and 36.3%, respectively), whereas tetranucleotide, pentanucleotide, and hexanucleotide motifs represented <10% of the markers. The motif AG/CT was the most abundant, accounting for 28.74% of the markers. One hundred EST-SSR markers (97 SSRs located in genes encoding transcription factors and 3 SSRs in genes encoding cellulose synthases) were amplified using polymerase chain reaction for detecting 24 ramie varieties. Of these 100 markers, 98 markers were successfully amplified and 81 markers were polymorphic, with 2–6 alleles among the 24 varieties. Analysis of the genetic diversity of all 24 varieties revealed similarity coefficients that ranged from 0.51 to 0.80. The EST-SSRs developed in this study represent the first large-scale development of SSR markers for ramie. These SSR markers could be used for development of genetic and physical maps, quantitative trait loci mapping, genetic diversity studies, association mapping, and cultivar fingerprinting.

## Introduction

Ramie (*Boehmeria nivea*), popularly called “China grass,” is a perennial diploid (2n = 28) herbaceous plant that belongs to the family *Urticaceae*. It is one of the most important natural fiber crops. Ramie fibers, which are stripped from the stem bast, are smooth, long, and have excellent tensile strength. This high fiber quality is the major reason ramie is widely cultivated in China, India, and other Southeast Asian and Pacific Rim countries. In China, ramie is the second most important fiber crop, with its growth acreage and quantity of fiber production being second only to those of cotton.

Because of the high economic potential of ramie, high fiber yield and excellent fiber quality are the main goals in ramie breeding initiatives. However, improvement of fiber traits has been severely hindered by the poor understanding of the genetic basis of fiber traits. This limited knowledge is largely due to the lack of specific genetic maps and quantitative trait loci (QTLs) for fiber-related traits. Recently, some markers that are not specific for location, such as sequence-related amplified polymorphism (SRAP), random amplified polymorphic DNA (RAPD), and inter simple sequence repeat (ISSR) markers, were used to analyze the genetic diversity in ramie [1]–[3]. However, these markers have the common shortcomings of poor repeatability and dominance. An alternative type of marker–simple sequence repeat (SSR) markers–has the following advantages: these markers are located at specific locations in the genome; they can be detected with high reproducibility; and they are multiallelic, codominant, analytically simple, and readily transferable [4], [5]. Therefore, SSR markers have been widely applied in the characterization and certification of plant materials, identification of varieties with agronomic potential, genetic mapping, and in crop-breeding programs [6]–[9]. To date, fewer than 100 SSR markers have been identified for ramie, including the markers generated for SSR-enriched genomic libraries and the expressed sequence tags (ESTs) deposited in public databases [10], [11].

Depending on the origin of the sequences used for the initial identification of SSRs, SSRs are classified as either genomic SSRs (derived from random genomic sequences) or EST-SSRs (derived from ESTs). Whereas genomic SSRs are not necessarily expected to either have genetic function or be closely linked to transcribed regions of the genome, EST-SSRs are tightly linked with functional genes that may influence certain important agronomic characters. Identification of SSRs has usually involved large-scale sequencing of the genome, the SSR-enriched parts of the genome, or EST libraries. However, this process is expensive, laborious, and time consuming. Next-generation sequencing technologies have enabled rapid identification of SSR loci derived from ESTs, which can be identified in any emergent species [12]–[14]. Our previous study, wherein we used sequencing and *de novo* assembly via Illumina paired-end sequencing, provided the first report of the ramie transcriptome [15]. The raw sequencing data from that study were deposited in the NCBI Sequence Read Archive (accession number SRA057664), and 43,990 ramie EST sequences were identified [15]. In the present study, these 43,990 ESTs were used to detect SSRs for the large-scale development and characterization of SSR markers. Development of SSR markers will facilitate genetic and genomic studies of ramie.

## Materials and Methods

### Plant Materials and DNA Extraction

Twenty-four ramie accessions collected from 9 provinces of China were used for the polymorphic analysis of SSR markers (Table 1). All 24 varieties were grown in the experimental fields of the Institute of Bast Fiber Crops, Chinese Academy of Agricultural Sciences, Changsha, China. Fresh leaves of each variety were collected for DNA extraction according to the cetyltrimethyl ammonium bromide (CTAB) method [16].

**Table 1:** Accessions used for diversity analysis.

Accessions	Abbreviation	Origin	Cluster	Accessions	Abbreviation	Origin	Cluster
Qingyezhuma	QZM	Hainan, China	I	Wayaozhuma	WZM	Guangxi, China	I
Zhongzhu 1	ZZ1	Guangxi, China	I	Gangza 2	GZ2	Jiangxi, China	I
Hejiangqingma	HQM	Sichuan, China	III	Miaobazhuma	MZM	Chongqing, China	I
Madarentuma	MTM	Hunan, China	I	Simaohongzhuma	SZM	Yunnan, China	II
Bijieqingma	BQM	Guizhou, China	I	Shuiqingqingma	SQM	Guangxi, China	II
Pingguangbaima	PBM	Guizhou, China	II	Yichuntongpichun	YTC	Jiangxi, China	I
Hongganma	HGM	Guangxi, China	II	Yachibaima	YBM	Guizhou, China	I
Longtanbaima	LBM	Sichuan, China	I	Rongchangzhuma	RZM	Chongqing, China	I
Jinxiqingma	JQM	Guangxi, China	I	Qingpima	QPM	Sichuan, China	I
Yichunjigubai	YGB	Jiangxi, China	I	Shizhuanbaiganma	SBM	Shanxi, China	I
Boyangqingyema	BYM	Jiangxi, China	I	Liangjiangjiama	LJM	Sichuan, China	I
Hongpi 1	HP1	Sichuan, China	I	Fenyiqingyema	FQM	Jiangxi, China	I

### Identification of SSR Loci and Development of Markers

Mining for putative SSRs was performed using the AutoSSR software [17]. The default criteria were used to select a minimum of 8 repeats for dinucleotide motifs, 6 repeats for trinucleotide motifs, 5 repeats for tetranucleotide motifs, and 4 repeats for pentanucleotide and hexanucleotide motifs. The EST sequences were used to design primers flanking the putative SSRs. Input criteria for the Primer 3.0 software for designing primers [18] were as follows: length, 17–23 bp; GC content, 40–60%; and estimated amplicon size, 100–300 bp.

### Classification of Cluster of Orthologous Groups (COG) Functions

All EST sequences that contained an SSR motif were classified into eukaryotic COGs categories according to the results of BLASTX searches against amino acid sequences in the COG data set (http://www.ncbi.nlm.nih.gov/COG/) [19]. These sequence similarities were judged to be significant when the E-value was less than 1E –10.

### Amplification of SSR-containing Regions and Detection of Polymorphisms

One hundred SSR markers (Table S1) selected for genotyping of 24 ramie varieties were amplified as previously described [10], and the SSR assay was carried out as described by Wu and Tanksley [20].

### Determination of Genetic Relationships among 24 Ramie Accessions

To assess the usefulness of the SSR primer pairs developed in this study, we analyzed the genetic relatedness among the 24 ramie accessions by using these SSR markers. The allelic data were converted into a binary matrix, with the scores 1 and 0 denoting the presence or absence of a given allele, respectively. The data were analyzed using the Numerical Taxonomy Multivariate Analysis System (NTSYS-pc) version 2.10 software [21]. Genetic similarity (GS) coefficients were calculated based on the coefficient for similarity matching by using the SIMQUAL module of the software. Using the GS matrix, we constructed a dendrogram by the unweighted pair group method with arithmetic average (UPGMA) to determine genetic relationships among the 24 genotypes.

## Results

### Development of SSR Markers

**Figure 1:**
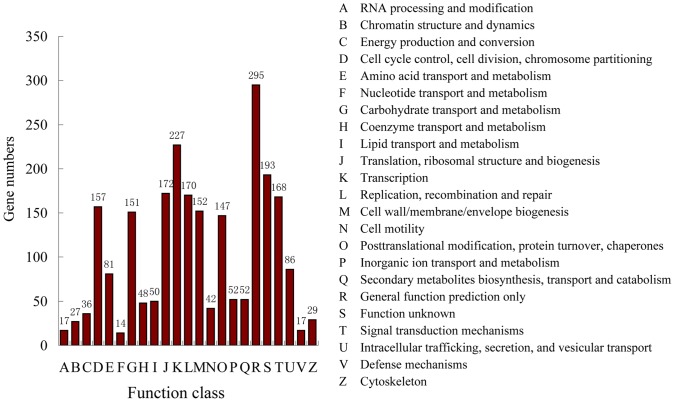
Functional classifications of ESTs that contain SSRs, based on COG searches.

A total of 43,990 EST sequences with a total size of 36.26 Mb were used to detect SSR loci by using the AutoSSR software (Table 2); 1,878 SSR loci were identified in 1,685 of the 43,990 EST sequences (Table 2). This shows that of the 43,990 ESTs, 3.83% contained at least 1 SSR. The frequency of occurrence for EST-SSRs was 1 SSR per 19.3 kb of EST sequence. The functions of the ESTs that contained SSRs were classified according to COG, and 1,685 sequences were assigned to 23 COG functional categories (Figure 1). Among the 1,685 ESTs examined, 127 sequences contained 2 SSR loci, 6 sequences contained 3 SSR loci, and 1 sequence contained 4 SSR loci. In addition, 49 ESTs contained SSRs that were present in compound formation with several SSR motifs. Finally, 1,827 primer pairs complementary to sequences that flank SSR regions were designed for identifying the SSR markers (Table S2).

**Table 2:** Results of searches for EST-SSRs.

Search item	Numbers
Total number of sequences examined	43,990
Total size of sequences examined (bp)	36,261,285
Total number of SSRs identified	1,878
Total number of SSR markers developed	1,827
Number of SSR-containing sequences	1,685
Number of sequences containing more than 1 SSR	134
Marker number of SSRs present in compound formation	49
Dinucleotides	665
Trinucleotides	663
Tetranucleotides	96
Pentanucleotides	178
Hexanucleotides	176

### Characterization of SSR Markers

Next, we analyzed the frequencies, types, and distributions of the 1,827 SSRs amplified using the primers designed in this study. For these 1,827 markers, dinucleotide and trinucleotide repeat motifs were the most abundant types (665 [36.4%] and 663 [36.3%], respectively) (Table 2). Only 96 tetranucloetide, 178 pentanucleotide and 176 hexanucleotide SSR markers were identified (Table 2). In addition, 49 markers contained a compound motif (Table 2). The dinucleotide to hexanucleotide motifs were further analyzed to characterize the SSR length (or number of repeat units, Table 3). The lengths of most (77.6%) SSRs ranged from 16 to 20 bp, followed by those in the 21 to 24 bp length range (372 SSRs, 19.8%). Twenty-two SSRs were longer than 30 bp.

**Table 3:** Length distribution of EST-SSRs based on the number of repeat units.

Number ofrepeat units	Dinucleotides	Trinucleotides	Tetranucleotides	Pentanucleotides	Hexanucleotides
4				164	174
5			89	27	7
6		527	14	0	4
7		137	0	0	3
8	412	21	0	0	1
9	205	0	0	1	0
10	60	1	0	0	0
11	20	0	0	1	1
12	6	0	1		
≥13		2			

**Figure 2:**
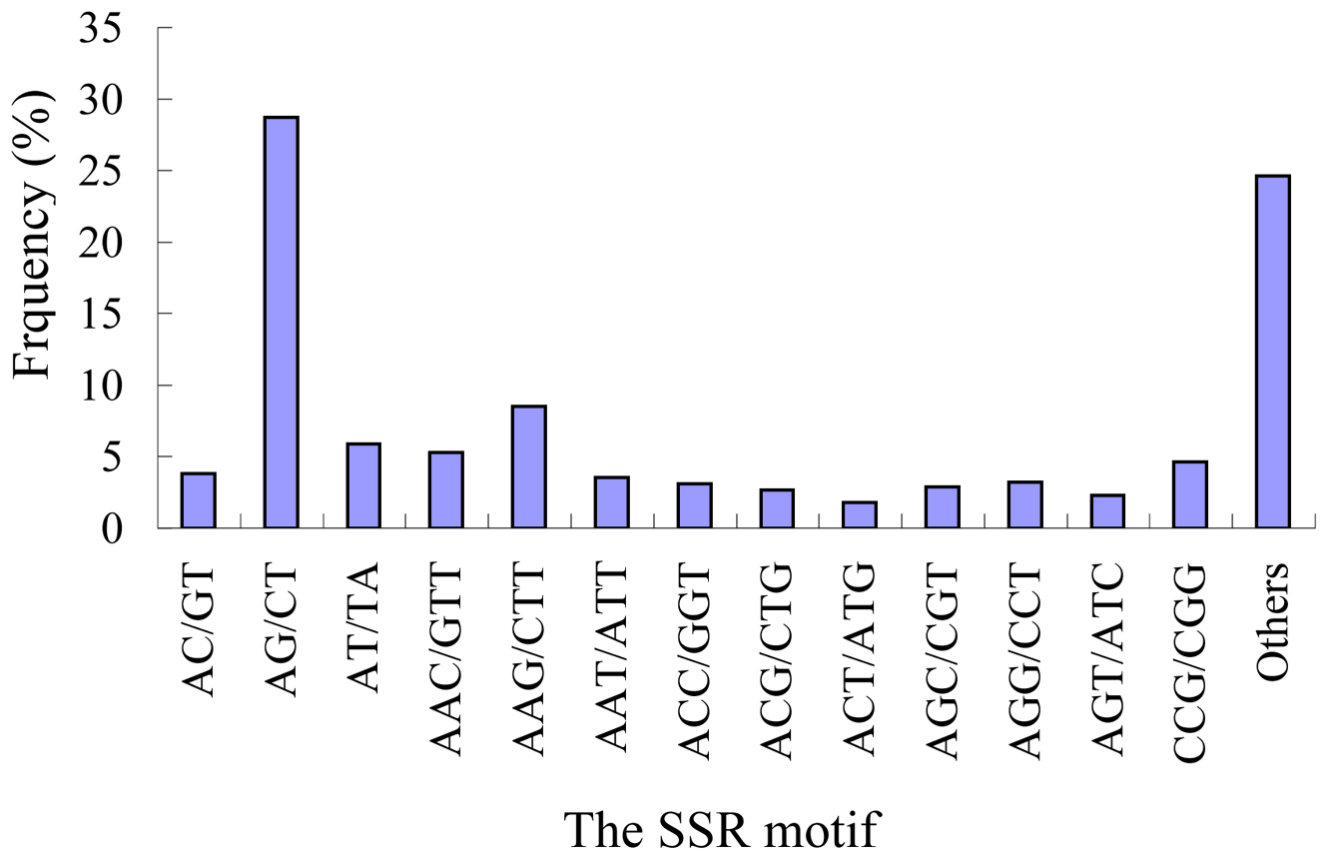
Frequency distribution of EST-SSRs based on motif sequence types.

Within the developed SSR markers, 151 motif sequence types were identified. Among these, there were 3, 10, 18, 44, and 76 motifs containing dinucleotide, trinucleotide, tetranucleotide, pentanucleotide, and hexanucleotide repeats, respectively. The AG/CT dinucleotide repeat was the most abundant motif detected in the SSRs (525, 28.74%), followed by the motifs AAG/CTT (156, 8.54%), AT/TA (108, 5.91%), AAC/GTT (97, 5.31%), CCG/CGG (85, 4.65%), AC/GT (70, 3.83%), AAT/ATT (65, 3.56%), AGG/CCT (59, 3.23%), ACC/GGT (57, 3.12%), AGC/CGT (53, 2.90%), ACG/CTG (49, 2.68%), AGT/ATC (42, 2.30%), and ACT/ATG (33, 1.81%) (Figure 2). The remaining 131 types of motifs accounted for 24.63% of all the SSRs analyzed (Figure 2).

### Analysis for Detection of Polymorphism in the SSR Markers

**Figure 3:**
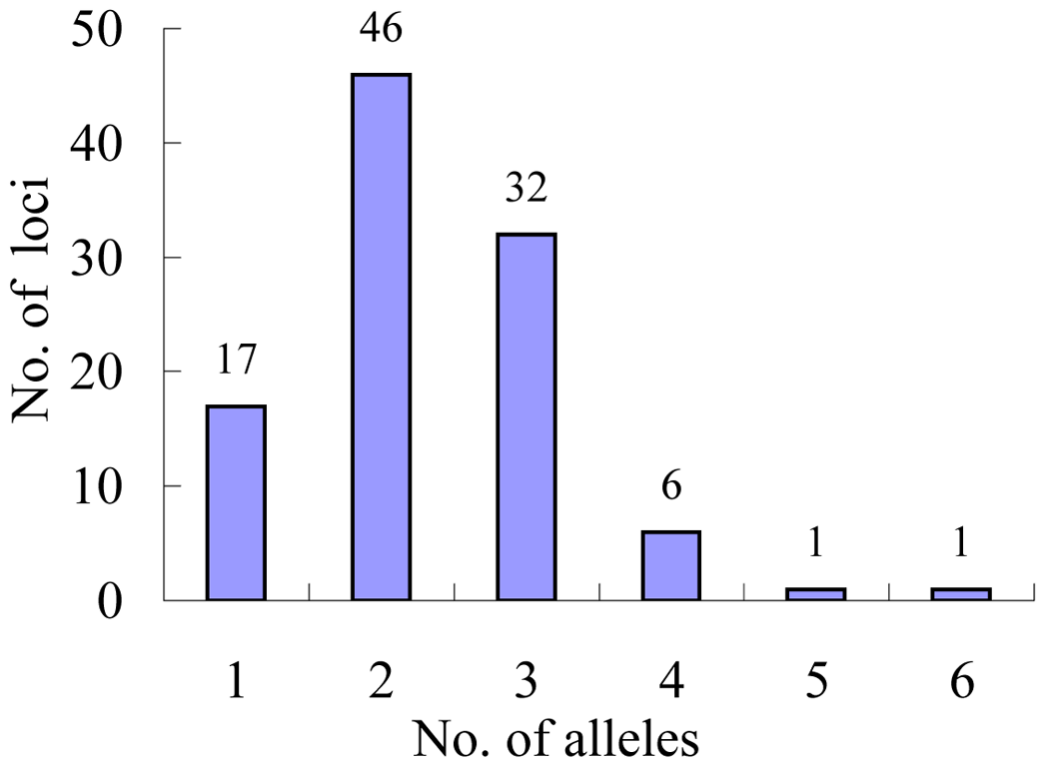
Distribution of the number of alleles per locus.

**Figure 4:**
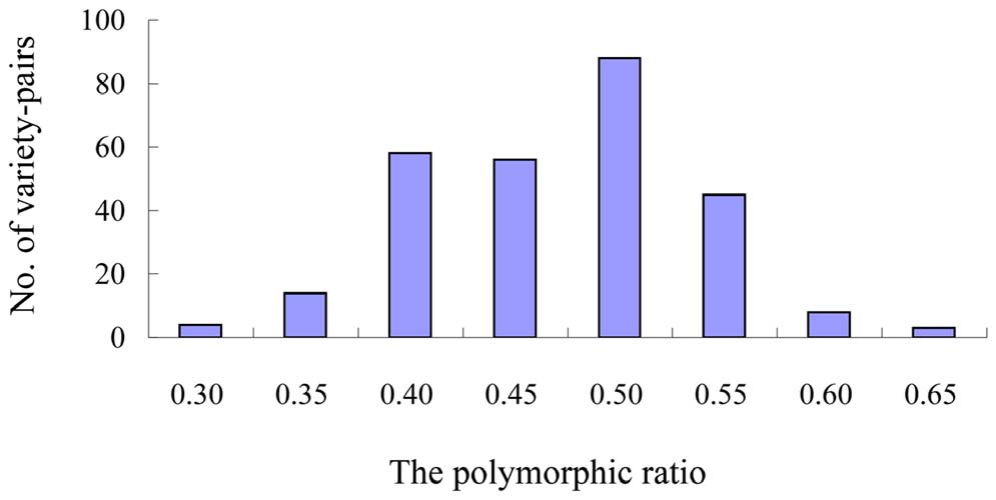
Distribution of the polymorphic ratio of variety pairs.

To assess the quality of the SSR markers for detection of 24 ramie varieties, 97 SSRs located in genes that encode transcription factors (TFs) and 3 SSRs located in genes that encode cellulose synthases (CesAs) were amplified using polymerase chain reaction (PCR) (Table S1). Successful amplification was achieved for 98 primer pairs. Three SSRs (RAM0590, RAM0667, and RAM0818) and 1 SSR (RAM0787) were amplified at 2 and 3 loci, respectively. Among the 103 loci that were successfully amplified by the 98 SSR markers, 46, 32, 6, 1, and 1 loci had 2, 3, 4, 5, and 6 alleles, respectively, in the 24 varieties, whereas the remaining 17 loci showed no polymorphism among the 24 ramie varieties (Figure 3). Considering 2 varieties as a variety pair, 276 variety pairs were found among the 24 varieties. Whereas 247 variety pairs (89.5%) showed polymorphisms with the ratio ranging from 35% to 55%, 18 and 11 variety pairs (6.5% and 4.0%, respectively) showed a polymorphic ratio less than 35% and more than 55%, respectively (Figure 4). Among 276 variety pairs, the largest polymorphic ratio (62%) was observed between SZM and YTC and the smallest polymorphic ratio (27%) was observed between QZM and ZZ1 (Table S3).

### Evaluation of Genetic Relationships among 24 Ramie Varieties

**Figure 5:**
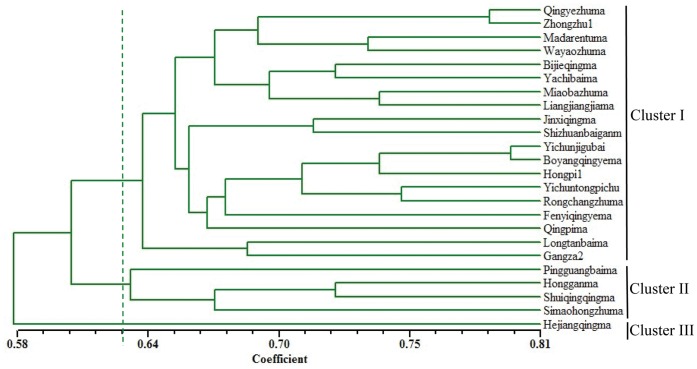
Dendrogram plot for 24 ramie varieties based on cluster analysis of 81 polymorphic EST-SSR markers.

A total of 223 SSR bands from 81 polymorphic primer pairs were used to evaluate genetic diversity and relatedness among the 24 ramie genotypes. Similarity coefficients were used to examine their genetic relationships. All possible genotypes showed similarity coefficients ranging from 0.51 to 0.80. The smallest similarity coefficient (0.51) was observed between SZM and YTC, and the largest similarity coefficient (0.8) was found for 2 local varieties YGB and BYM, which originated from the Jiangxi Province, China. Taking a GS score of 0.62 as the threshold, the 24 ramie accessions could be distinctly classified into 3 clusters (Table1, Figure 5). Cluster I was the major group comprising 19 varieties and cluster II comprised 4 varieties, PBM, HGM, SZM, and SQM. Cluster III comprised only 1 variety, HQM.

## Discussion

### Development of 1,827 EST-SSR Markers for Ramie

The current shortage of SSR markers is a major obstacle for genetic and breeding studies in ramie. Fewer than 100 SSR markers have been reported in ramie [10], [11], which is far from sufficient for effective genetic mapping and marker-assisted breeding. The lack of a genetic map for ramie and the absence of QTLs for agronomically important traits have resulted in a large gap in the understanding of the genetic basis for the desirable features of ramie.

In this study, we identified 1,827 EST-SSR markers based on ramie ESTs that had been assembled in our previous transcriptome sequencing study [15]. A few instances of primer mismatch occurred, and in such cases, some EST-SSRs might have failed to amplify because the primers were designed across splice sites or because large introns were present within the target amplicon [13], [22]–[24]. In order to evaluate the quality of the EST-SSR primers designed in this study, 100 SSRs were amplified, and 98 primer pairs successfully amplified their target sequences (98% success rate). Given that 100 EST-SSR markers were chosen for amplification according to the function of the gene in which the SSR was located (i.e., in a gene that encodes either a TF or CesA), and the functions of the specific genes did not influence the PCR amplification of the marker, the 98% success observed for the amplification could be extrapolated to mean that 1,827 markers can be successfully amplified using PCR. In addition, the polymorphism analysis showed that 81 of 98 SSRs had polymorphisms with 2–6 alleles among the 24 ramie varieties. The 81 polymorphic EST-SSR markers permitted elucidation of the genetic diversity and relationships among the 24 ramie genotypes. These results suggest that the EST-SSR markers developed in this study are of excellent quality. To our knowledge, the present study represents the first successful development of SSR markers in ramie on a large scale. These markers will provide a valuable resource for future genetic mapping, QTL analysis, comparative genetic studies, and molecular marker-assisted selection breeding (MAS).

### EST-SSR Frequency and Distribution

Among 43,990 ramie ESTs, approximately 3.83% contained SSRs. This frequency is higher than those reported for grapes (2.5%) [6], flax (3.5%) [24], and barley (2.8%) [22], but lower than those reported for coffee (18.5%) [25] and wheat (7.41%) [26]. The average distance (in kb) between 2 EST-SSRs is 3.4 in rice, 5.46 in wheat, 6.3 in barley, 7.4 in soybean, 8.1 in maize, 11.1 in tomato, 13.8 in *Arabidopsis*, 14 in poplar, 16.5 in flax, and 20 in cotton [23]–[27]. The observation that the 19.3-kb interval found for the ramie EST-SSRs suggests that EST-SSRs are less prevalent in ramie than in other plant species. Dinucleotide and trinucleotide motifs were the 2 most abundant motifs (36.4% and 36.3%, respectively), and this finding was in agreement with the EST-SSR distribution that has been reported in peach, pumpkin, coffee, spruce, and kiwi fruit [25], [28]–[31]. However, the number of EST-SSRs, the average distance between EST-SSRs, and the abundance of dinucleotide and trinucleotide motifs are all highly dependent on the use of different SSR search criteria, the size of databases, and the database mining tool used [9], [25].

AG/CT was the most abundant motif and accounted for 28.74% of all markers; this frequency was similar to that observed in sweet potato [13]. Among the trinucleotide motifs, AAG/CTT was the most abundant, with a frequency of 8.54%. Interestingly, no SSR of the GC/CG motif was detected in the 43,990 ESTs analyzed. The abundance of CCG/CGG motifs was reported to be a specific feature of monocot genomes [26]. It appears that GC-rich SSR motifs are more frequent in ESTs from monocots than in those from dicots, where AG/CT and AT/AT were the most frequent dinucleotide motifs, and CTT/AAG was the most frequent trinucleotide motif [6], [24], [27], [29]–[31]. Thus, the results obtained in ramie were corroborated by other studies.

### Potential Application in Ramie Breeding

Changes in the lengths of SSRs might affect gene function when the EST-SSR is located in a protein-coding region. Although trinucleotide and hexanucleotide SSRs do not cause frame shifts when present in ESTs because they are found in multiples of 3 (i.e., the number of nucleotides in a codon), the insertion or deletion of a trinucleotide and hexanucleotide motif can cause several changes in the primary structure of a protein, such as substitution, insertion, or deletion of amino acids. Moreover, the length changes due to dinucleotide, tetranucleotide, and pentanucleotide motifs SSRs are likely to cause frame shifts, which can disrupt the function of the protein encoded by the gene in which the SSR occurs. When genes that contain SSRs influence agronomically important characteristics, the SSR in the protein-encoding region can be developed as a functional marker for ramie breeding. Even if the EST-SSRs are located in the 5′- and 3′-untranslated regions (UTRs) of any gene, these SSRs will be tightly linked with functional genes. These SSR markers will therefore be useful for selecting and pyramiding agriculturally valuable alleles in ramie MAS.

TFs play a key role in regulating gene expression at the mRNA level and regulate many biologically important processes such as progression through the cell cycle, maintenance of metabolic and physiological homeostasis, and responses to environmental stimuli [32]. In this study, 97 EST-SSRs were found in the coding regions of TFs. Of these 97 EST-SSRs, 79 were polymorphic, with 2–6 alleles among the 24 ramie varieties analyzed (Table S1). Association analysis between EST-SSR markers and traits will probably be useful for identifying how these TFs influence agronomically important traits.

## Supporting Information

Table S1 **Hundred EST-SSR markers used for PCR amplification.**
(XLS)

Table S2 **The information of 1827 EST-SSR markers developed.**
(XLS)

Table S3 **The polymorphic ratios among 24 ramie varieties.**
(XLS)
